# Assessment of Fall Risk in Oncology Patients: A Scale Development Study

**DOI:** 10.1111/scs.70221

**Published:** 2026-03-23

**Authors:** Sibel Serçe, Soner Berşe, Özlem Ovayolu, Nimet Ovayolu

**Affiliations:** ^1^ Faculty of Health Science, Department of Nursing Gaziantep Gaziantep University Gaziantep Turkey; ^2^ Faculty of Health Science, Department of Nursing Gaziantep SANKO University Gaziantep Turkey

**Keywords:** fall risk, oncology nursing, oncology patients, scale development

## Abstract

**Objective:**

This methodological study aimed to develop and validate a reliable scale for assessing fall risk in oncology patients.

**Methods:**

The research included 415 patients (217 for Exploratory Factor Analysis [EFA], 198 for Confirmatory Factor Analysis [CFA]) from a university hospital's oncology clinic and polyclinic. Data were collected using a demographic questionnaire and a draft Fall Risk Assessment Scale for Oncology Patients.

**Results:**

EFA revealed a single‐factor structure consisting of 19 items, explaining 79.34% of the total variance. The scale demonstrated high internal consistency (Kuder–Richardson 20 = 0.964). CFA further confirmed the single‐dimensional model, indicating robust construct validity.

**Conclusion:**

The newly developed scale provides a valid and practical tool for identifying fall risk among oncology patients. Its implementation may support the identification of individuals at higher fall risk in oncology settings and inform clinical decision‐making and preventive care planning.

## 1 Introduction

Individuals diagnosed with cancer are a sensitive group that needs to be specially addressed in terms of fall risk due to the disease process and the side effects of the treatments applied [1, 2]. Falls in these patients can occur due to intrinsic and extrinsic factors throughout the treatment process. Intrinsic factors that may lead to falls include the presence of tumours affecting balance, depression, anaemia, fatigue, side effects of treatment, increased medication use, and restricted physical activity. Extrinsic factors include uneven walking surfaces, inadequate lighting, rugs with tassels, and slippery floors [3].

The literature particularly highlights hematologic malignancies, brain tumours or metastases, anaemia, chemotherapy‐related complications, and prolonged hospital stays as significant causes of falls among oncology patients [4]. Additionally, falls in oncology patients, even if they do not cause any injury, can lead to fear of falling again, anxiety, avoidance of physical activity, and depression. Consequently, patients may experience extended hospital stays, increased medication use, restricted activity, and a decrease in quality of life [2]. Particularly in geriatric individuals, it has been found that 50% of those who fall subsequently avoid activities due to fear, and thus, their independence and quality of life are significantly affected, as they refrain from activities such as travel, shopping, bathing, dressing, cleaning, and cooking [5]. In a study by Savvakis et al., it was determined that increasing physical activity, strength, balance, and mobility in geriatric individuals is effective in reducing the fear of falling [6]. Choi et al. emphasised that patients need to perceive the factors causing fall risk accurately, and nurses should assess these perceptions and inform patients about precautions to reduce fall risk [7]. Interventions aimed at preventing falls in cancer patients and environmental adjustments to prevent falls at home after discharge have been reported to reduce the rate of falls, increase knowledge about fall prevention, and raise fall awareness [2].

In managing falls, it is crucial to identify high‐risk patients and implement particular preventive interventions with multidisciplinary support [5]. Therefore, it is essential to identify risk factors early and plan effective interventions specific to each risk factor [8]. A study by Chari et al. evaluated the fall risk of hospitalised individuals and reduced the risk by 40% [9]. The study by Kolb et al. found that patients with Chemotherapy–Induced Peripheral Neuropathy symptoms had an approximately three times higher risk of falling than those without symptoms [10]. In a meta‐analysis of cancer patients receiving inpatient treatment, it was reported that treatment‐related factors such as opioids, benzodiazepines, sedative medications, and radiotherapy increased the risk of falls [11]. Tools are clearly needed to assess fall risk and identify potential risk factors early in patients undergoing cancer treatment [8]. To review the existing literature, searches were conducted in PubMed, Scopus, Web of Science, and the Turkiye National Citation Index databases using the keywords fall risk scale, ‘cancer patients’, ‘oncology’; the search covered the years 2000–2022 and included articles published in English or Turkish. Publications related to fall risk assessment scales were evaluated in terms of studies covering adult and paediatric oncology patients, and a total of 45 scales were identified. These scales, including the Morse Fall Scale, Hendrich II, and ITAKI Fall Risk Scale, which are widely used in clinical practice, were compared in terms of applicability, sensitivity, specificity, and psychometric properties in different populations. Although numerous fall risk assessment tools exist for both adult and paediatric patients ([12, 13, 14, 15, 16, 17]), these tools may produce varying results across different patient populations [8].

It has been reported that some general scales used in the literature to assess the risk of falling do not adequately reflect patient characteristics and risk factors in certain clinical settings and that measurement accuracy may therefore be limited. In line with these limitations, fall risk scales specific to certain clinical settings, such as rehabilitation hospitals and acute care environments, have been developed, and the validity and reliability of these scales have been established through methodological studies [18, 19]. The fundamental rationale for developing scales specific to the clinical setting is that the patient profile, treatment processes, functional status and environmental conditions differ significantly from the general patient population. A systematic review study also shows that there are numerous fall risk assessment tools in the literature, that these tools however perform differently depending on the clinical setting and that scales developed for specific patient groups yield more accurate results in risk classification [20]. These findings demonstrate that the scale development approach for specific patient groups and care settings is a methodologically robust approach that is accepted in the literature.

The Morse Fall Scale is widely used in clinical practice [21]. However, fall risk assessment instruments such as the Hendrich II [22] have been reported to exhibit psychometric limitations, particularly when applied to oncology patients. Furthermore, the literature indicates that the predictive accuracy of current fall risk assessment tools used in oncology patients may be limited; some studies have reported that sensitivity and specificity values fall below the desired level, which may increase the risk of misclassification [23]. The main reason for this situation is that tools that assess the risk of falling do not sufficiently cover oncology‐specific risk factors such as chemotherapy‐related neurotoxicity, anaemia, balance loss due to metastasis, sedative use, and prolonged hospitalisation [10, 11, 24, 25, 26]. Therefore, since current general fall risk scales cannot fully reflect the complex and variable clinical condition of oncology patients, merely modifying or adding items may compromise the scale's validity and reliability. Therefore, the direct application of existing tools to oncology patients is limited and cannot be easily adapted.

In certain national healthcare systems, the ITAKI Fall Risk Scale, developed by the Ministry of Health, is widely used (TC. Sağlık [8, 27]). However, this scale does not sufficiently account for the specific risk factors related to falls in oncology patients [28]. A review of national and international studies supports the importance and necessity of developing a valid and reliable fall risk assessment tool that accurately reflects the clinical characteristics and specific risk factors of oncology patients. Therefore, this study aims to develop a fall risk assessment scale for oncology patients and to perform validity and reliability analyses of this scale. The aim is to identify the factors contributing to falls among oncology patients, to develop strategies for fall prevention, and to support caregivers and healthcare professionals in implementing appropriate precautionary measures, particularly for individuals at high risk.

## 2 Materials and Methods

### 2.1 Study Design

This methodological study aimed to develop a reliable and valid tool for assessing fall risk in oncology patients.

### 2.2 Study Setting and Duration

After obtaining the necessary approvals, the study was conducted between August 1, 2022, and August 1, 2023, with patients in the oncology clinic and polyclinic of a university hospital.

### 2.3 Inclusion Criteria

The study included patients diagnosed with cancer who were being followed in the oncology clinic or polyclinic, had no communication difficulties, and voluntarily agreed to participate. Patients who did not meet these criteria were excluded from the study.

### 2.4 Study Population and Sample

In research involving scale development, it is recommended that the sample size be five to 10 times larger than the number of items on the scale [29, 30]. Accordingly, 217 patients from the adult oncology clinic and polyclinic were included in the preliminary analysis and structural examination of the 43‐item draft scale. In comparison, 198 patients were included in the Confirmatory Factor Analysis (CFA).

### 2.5 Development Stages of the Fall Risk Assessment Scale for Oncology Patients

#### 2.5.1 Determining the Draft Scale Items

A literature review was conducted to develop the fall risk assessment scale for oncology patients. National and international fall risk assessment tools were reviewed, and based on the findings, an item pool and a 48‐item draft scale were created.

#### 2.5.2 Content Validity

Content validity refers to how well a scale and its items fulfil their intended purpose [31, 32, 33]. The content validity of the draft scale was assessed using the Lawshe technique, with input from 10 experts in the field [34]. Items that did not meet the content validity criterion (below 0.80) were eliminated, resulting in a reduction to 43 items. The content validity index of the draft scale was calculated to be 0.92.

#### 2.5.3 Pilot Application

The final version of the draft scale was piloted with 50 patients not included in the primary sample. The Kuder–Richardson value and the clarity of the items were evaluated. Based on participants' feedback, minor adjustments were made to some of the wording.

#### 2.5.4 Exploratory Factor Analysis and Confirmatory Factor Analysis

The draft scale was applied to separate sample groups (a sample group five to ten times the size of the items) for Exploratory Factor Analysis (EFA) and CFA [30, 35]. The 43‐item draft scale was administered to a sample group of 217 individuals for EFA, and 24 items with low factor loadings were removed, resulting in a 19‐item unidimensional scale. It was then validated using CFA by applying it to an independent sample group of 198 individuals. These analyses demonstrated that the scale validly and reliably measures the risk of falls in oncology patients.

#### 2.5.5 Pilot Application and Analysis Results

The scale was administered to 50 patients who participated in the pilot application but were not included in the sample using the face‐to‐face interview method. The purpose of this application was to evaluate the understandability of items and calculate the Kuder–Richardson 20 (KR‐20) reliability coefficient. The patients were asked whether they had difficulty understanding each item during the application. Thus, the items were evaluated in terms of language clarity, and minor adjustments were made to the wording of the questions based on the feedback received.

The KR‐20 reliability coefficient calculated at the end of the pilot study indicates that the items measure the same construct in a consistent manner. This stage is only a preliminary testing process for the scale draft and was not used to assess construct validity.

Additionally, the study was designed in two phases. First, the scale structure was established by applying EFA with a sample of 217 individuals. In the second stage, structural equation modelling was applied to a sample of 198 independent individuals to validate the structure. Thus, by testing the structure on different samples, it was clearly determined that the scale did not have a split structure.

### 2.6 Measuring Instruments

Data were collected through face‐to‐face interviews using the Fall Risk Assessment Scale for Oncology Patients (FRAS‐OP), a questionnaire form containing questions about patients' sociodemographic characteristics and disease‐related information.

The pilot group of 50 individuals was used only during the preliminary testing phase of the FRAS‐OP to assess item clarity and internal consistency and was not included in the main analysis.

#### 2.6.1 Questionnaire

This form, prepared by the researchers based on the literature, included 25 questions about the patient's sociodemographic characteristics and disease status [2, 4, 6, 7, 36].

#### 2.6.2 Fall Risk Assessment Scale for Oncology Patients

The psychometric properties of the FRAS‐OP were evaluated through EFA and CFA to assess construct validity. Internal consistency reliability was examined using the KR‐20 coefficient. Detailed results of these analyses are presented in the ‘Results’ section.

### 2.7 Ethical Aspects of the Research

The research process was conducted in accordance with the ethical principles of the Declaration of Helsinki. Institutional approval was obtained from the Gaziantep University Clinical Research Ethics Committee (protocol no: 2022/213) for the research, as well as from the Oncology Clinic and Outpatient Clinics of Gaziantep University Şahinbey Research and Application Hospital, where the research was conducted. Before starting the research, the participants were informed about the research, and their written and verbal consent was obtained.

### 2.8 Data Analysis

To evaluate the data obtained in this study, EFA was first conducted to examine the construct validity of the scale [37, 38, 39]. The suitability of the dataset for factor analysis was assessed using the Kaiser–Meyer–Olkin (KMO) measure and Bartlett's test of sphericity. Factor loadings were determined using the Varimax rotation method.

Subsequently, CFA was performed using the AMOS software to verify the factor structure obtained from EFA. Model fit was evaluated using a range of goodness‐of‐fit indices, including the chi‐square to degrees of freedom ratio (CMIN/df), Normed Fit Index (NFI), Incremental Fit Index (IFI), Comparative Fit Index (CFI), Root Mean Square Error Approximation (RMSEA), and Goodness‐of‐Fix Index (GFI). The internal consistency reliability of the scale was assessed using the KR‐20 coefficient.

### 2.9 Confirmatory Factor Analysis (CFA)

Confirmatory Factor Analysis was conducted using AMOS to test the factor structure obtained from Exploratory Factor Analysis. Model fit was assessed using a stepwise approach. First, the initial measurement model was tested without any modifications. The chi‐square/df ratio (CMIN/df), Normalised Fit Index (NFI), Incremental Fit Index (IFI), Comparative Fit Index (CFI), Root Mean Square Error of Approximation (RMSEA), and Goodness of Fit Index (GFI) were considered in the evaluation of model fit.

Following the evaluation of the initial model, the modification indices were examined. Only error covariances that can theoretically be justified between items under the same factor were taken into account. In line with these modification indices, limited adjustments were made to the models in order to improve model fit. Following these adjustments, the DFA was re‐estimated and the goodness‐of‐fit indices for the modified model were evaluated.

## 3 Results

### 3.1 Characteristics of Oncology Patients and Findings From EFA and CFA

The results of the EFA of the FRAS‐OP, the average age of the patients was 57.35 ± 13.04 years, with 62.7% being female, 58.3% having completed primary education, 84.8% being married, 61.8% having an income equal to expenses, and 65.9% living in the city. Additionally, 26.5% were receiving chemotherapy, 33.2% were receiving a combination of chemotherapy, surgery, and radiotherapy, 45.2% had a chronic disease, and 81% had not fallen in the past year. In comparison, 93.6% had not fallen in the past month.

For the CFA, the average age was 58.02 ± 12.59 years, with 53% being female, 58.6% having completed primary education, 91.9% being married, 52% having an income equal to expenses, and 53.5% living in the city. Furthermore, 42.9% were receiving chemotherapy, 19.7% were receiving a combination of chemotherapy, surgery, and radiotherapy, 47.5% had a chronic disease, and 77.3% had not fallen in the past year. In comparison, 82.4% had not fallen in the past month (Table 1).

**TABLE 1:** Comparison of exploratory and confirmatory factor analysis findings of the fall risk assessment scale for oncology patients.

Characteristics		EFA (*n*:217)		CFA (*n*:198)	
		*n*	%	*n*	%
**Age**	Mean ± SD	57.35 ± 13.047		58.02 ± 12.594	
**Gender**	Female	136	62.67	105	53.03
	Male	81	37.33	93	46.97
**Educational Level**	Illiterate	37	17.13	37	18.69
	Literate	10	4.63	16	8.08
	Primary Education	126	58.33	114	57.58
	High School	32	14.81	22	11.11
	University and above	11	5.09	9	4.55
**Marital Status**	Married	184	84.79	182	91.92
	Single	33	15.21	16	8.08
**Income Level**	Less than expenses	81	37.33	92	46.46
	Equal to expenses	134	61.75	103	52.02
	More than expenses	2	0.92	3	1.52
**Place of Residence**	City	141	65.89	106	53.54
	District	55	25.70	64	32.32
	Village	18	8.41	28	14.14
**Type of Treatment**	Chemotherapy	48	26.52	85	42.93
	Radiotherapy	3	1.66	3	1.52
	Surgery	11	6.08	7	3.54
	Surgery and Chemotherapy	29	16.02	35	17.68
	Radiotherapy and Chemotherapy	19	10.50	25	12.63
	Radiotherapy and Surgery	3	1.66	1	0.51
	All	60	33.15	39	19.70
	Other	8	4.42	3	1.52
**Presence of Chronic Disease**	Yes	98	45.16	94	47.47
	No	119	54.84	104	52.53
**Fall in the Past Year**	Yes	41	18.98	35	22.73
	No	175	81.02	119	77.27
**Fall in the Past Month**	Yes	14	6.45	18	17.65
	No	203	93.55	84	82.35

### 3.2 Reliability and EFA Findings of the FRAS‐OP

The KMO value of the developed scale was determined to be 0.943, and Bartlett's Test was found to be significant (*p* < 0.05). This indicates that the data are suitable for factor analysis and sufficient to identify the factors that cause the risk of falling in oncology patients.

In exploratory factor analysis, it was determined that the scale is unidimensional and consists of 19 items, and that this dimension explains 79.34% of the variance. This scale demonstrates that its single dimension encompasses the majority of the factors determining the risk of falling in oncology patients.

Factor loadings range from 0.666 to 0.951. This demonstrates that each item contributes meaningfully to the scale and that the scale can capture different but related dimensions of fall risk in oncology patients. Item‐total correlation coefficients ranged from 0.639 to 0.940, and the KR‐20 value was calculated as 0.964. This also demonstrates that the scale items consistently measure the risk of falling and can reliably distinguish between high‐ and low‐risk patients.

In confirmatory factor analysis, the structural model of the scale demonstrated acceptable, although borderline, fit indices, and model fit improved following theoretically justified modifications. These findings support the consistency of the 19‐item single‐factor structure with the observed data. Considering the clinical screening purpose of the instrument, the scale demonstrates acceptable psychometric properties and may be used as a supportive screening tool in clinical practice and research settings. The total score on the scale ranges from 19 to 38, with higher scores indicating increased fall risk, thereby enabling early identification of high‐risk patients and the planning of preventive interventions (Table 2).

**TABLE 2:** Reliability and exploratory factor analysis findings of the fall risk assessment scale for oncology patients.

Scale items	Item loadings	
1. Lives alone at home	0.886	
2. Underweight (Body Mass Index < 18.5 kg/m²)	0.838	
3. Overweight (Body Mass Index > 25 kg/m²)	0.928	
4. Alcohol consumption	0.666	
5. Smoking	0.849	
6. Wears glasses	0.887	
8. History of cerebrovascular disease	0.892	
9. Stage 1‐2 cancer patient	0.946	
11. Presence of bone metastasis	0.831	
12. Previous fall history	0.922	
13. Takes four or more medications	0.946	
14. Balance issues post‐chemotherapy	0.939	
15. Appropriate home environment adjustments	0.951	
16. Caregiver has adequate knowledge of the disease	0.900	
17. Assistance with bathing	0.893	
25. Experiences emotional changes	0.948	
26. Experiences dizziness	0.886	
27. Hand tremors	0.927	
29. Experiences drowsiness	0.845	
**Eigenvalue**	15.076	
**Explained Variance**	79.347	
**Total Variance %**	79.347	
**Kaiser‐Meyer‐Olkin Measure**	0.943	
**Bartlett's Test**	X^2^	7177.732
	df	171
	Sig	0.001
**Kuder‐Richardson**	0.964	

### 3.3 Correlation Coefficients of the FRAS Items for Adult Oncology Patients

The item‐total correlation coefficients for the FRAS‐OP ranged from 0.639 to 0.940 (Table 3). Item‐total correlation coefficients above 0.3 indicate strong internal consistency [40].

**TABLE 3:** Correlation coefficients of the fall risk assessment scale items for oncology patients.

Ölçek Maddeleri	*r*
1. Lives alone at home	0.872
2. Underweight (Body Mass Index < 18.5 kg/m^2^)	0.822
3. Overweight (Body Mass Index > 25 kg/m^2^)	0.916
4. Alcohol consumption	0.639
5. Smoking	0.832
6. Wears glasses	0.873
8. History of cerebrovascular disease	0.879
9. Stage 1–2 cancer patient	0.936
11. Presence of bone metastasis	0.812
12. Previous fall history	0.910
13. Takes four or more medications	0.935
14. Balance issues post‐chemotherapy	0.928
15. Appropriate home environment adjustments	0.940
16. Caregiver has adequate knowledge of the disease	0.886
17. Assistance with bathing	0.880
25. Experiences emotional changes	0.938
26. Experiences dizziness	0.873
27. Hand tremors	0.915
29. Experiences drowsiness	0.827

### 3.4 CFA Of the Fall Risk Assessment Scale for Oncology Patients

#### 3.4.1 Findings of Confirmatory Factor Analysis

In the CFA conducted to test the single‐factor 19‐item structure, it was determined that the fit indices for the initial model were acceptable but limited. It was observed that in the initial model, some fit indices did not fully meet the recommended threshold values and that the model did not therefore fit the data optimally.

Following the examination of modification indices, a limited number of error covariances were added between items that fall under the same factor and are theoretically meaningful. Following these limited adjustments, an improvement in model fit was observed. After modification, all standardised factor loadings in the model were found to be statistically significant. The RMSA value for this model was determined to be 0.087 and the GFI value 0.880, indicating that the model fit is acceptable but not perfect.

Although some compatibility indices fell slightly below the ideal cut‐off points, the scale's single‐factor structure was generally supported. Considering that the scale was developed for clinical screening purposes, the findings support the potential use of the scale in clinical applications and research settings (Table 4).

**TABLE 4:** Item/factor loading values of the fall risk assessment scale for oncology patients.

Relationships			Standardised regression weight	Regression weight	S.E.	*t*	*p*	AVE	CR
s1	<−	F1	0.738	1					
s2	<−	F1	0.645	1.005	0.109	9.195	0.001*		
s3	<−	F1	0.851	0.963	0.077	12.467	0.001*		
s4	<−	F1	0.662	0.897	0.095	9.457	0.001*		
s5	<−	F1	0.718	0.922	0.089	10.325	0.001*		
s6	<−	F1	0.762	1.033	0.094	11.027	0.001*		
s8	<−	F1	0.701	1.064	0.106	10.059	0.001*		
s9	<−	F1	0.869	0.984	0.077	12.765	0.001*	0.567	0.96
s11	<−	F1	0.652	0.983	0.106	9.3	0.001*		
s12	<−	F1	0.798	0.988	0.085	11.6	0.001*		
s13	<−	F1	0.839	0.899	0.073	12.27	0.001*		
s14	<−	F1	0.789	1.002	0.087	11.458	0.001*		
s15	<−	F1	0.792	1.029	0.09	11.502	0.001*		
s16	<−	F1	0.679	1.035	0.107	9.713	0.001*		
s17	<−	F1	0.773	1.057	0.094	11.192	0.001*		
s25	<−	F1	0.856	1.031	0.082	12.547	0.001*		
s26	<−	F1	0.697	1.081	0.108	10.001	0.001*		
s27	<−	F1	0.753	1.092	0.1	10.88	0.001*		
s29	<−	F1	0.666	1.065	0.112	9.516	0.001*		

* Indicates statistical significance at *p* < 0.001.

### 3.5 Reliability and Validity of the FRAS‐OP

Composite Reliability (CR) values were used to assess the scale's reliability. The Average Variance Extracted (AVE) values were calculated to evaluate the scale's convergent and discriminant validity. As per the literature, AVE values should be greater than 0.5, and CR values should be greater than 0.7 to establish convergent validity [41]. Table 4 shows the CR and AVE values for the factors in the FRAS for Adult Oncology Patients. The results demonstrate strong internal consistency and acceptable convergent validity, as CR values (0.96) exceeding the recommended threshold of 0.70 and AVE values (0.567) met the acceptable criterion and remained lower than the corresponding CR values.

### 3.6 Modification Indices of the FRAS‐OP

Modification indices were examined to improve model fit. Based on these indices and theoretical considerations, error covariances between items within the same factor and with related content (e.g., e2‐e20, e17‐e14, and e14‐e20) were allowed. Following these limited and theoretically justifiable modifications, the model fit indices improved and reached acceptable levels. The post‐modification fit indicated that the proposed model adequately fit the data. The single‐factor, 19‐item structure of the scale was supported, as presented in Table 5. The findings confirm the structure obtained from the EFA through confirmatory factor analysis using structural equation modelling.

**TABLE 5:** Modification index scores of the fall risk assessment scale for oncology patients.

Fit index	Post‐modification value	Acceptable fit	Good fit
CMIN/df	3.584	≤ 5	≤ 3
GFI	0.778	≥ 0.85	≥ 0.90
IFI	0.876	≥ 0.90	≥ 0.95
CFI	0.876	≥ 0.95	≥ 0.97
RMSEA	0.115	≤ 0.08	≤ 0.05
NFI	0.836	≥ 0.90	≥ 0.95

## 4 Discussion

Falls are common among cancer patients and can lead to severe injuries, disabilities, and even death. The literature indicates that cancer patients undergoing chemotherapy experience higher rates of falls compared to those not receiving chemotherapy [2]. This study found an increased risk of falls in geriatric patients (*p* < 0.05). Specifically, chemotherapy treatments in geriatric oncology patients are reported to increase the risk of falls by two to three times [3]. Therefore, identifying the factors associated with falls and implementing appropriate preventive strategies are important for maintaining patients' functional independence and quality of life.

Although the ITAKI Fall Risk Scale is widely used in Türkiye to assess fall risk, it was not applied as a criterion measure in the present study. Therefore, direct statistical comparisons or criterion validity analyses with ITAKI could not be conducted. Nevertheless, previous studies indicate that ITAKI primarily focuses on general clinical risk factors and may not sufficiently address oncology‐specific conditions such as chemotherapy‐related symptoms, combination therapies, or treatment‐related functional decline. A review of the literature also revealed that no scale has been specifically developed to assess fall risk exclusively in oncology patients. For this reason, the FRAS‐OP was developed, and its validity and reliability were evaluated.

The high KMO value and explained variance indicate a robust factor structure, consistent with methodological standards for scale development. The EFA revealed that the factor loadings ranged from 0.666 to 0.951, and the scale consisted of 19 items within a single‐factor structure explaining 79.34% of the total variance. The internal consistency of the scale, assessed using the Kuder–Richardson coefficient, was found to be 0.964, indicating very high internal consistency and strong reliability [29].

The items included in FRAS‐OP address oncology‐specific fall risk factors such as chemotherapy exposure, combination treatment protocols, advanced age, and the presence of chronic comorbidities. In this respect, FRAS‐OP differs from existing fall risk assessment tools that focus on general patient populations and provide a more systematic evaluation of risk specific to oncology patients. Similarly, Rahimi et al. emphasised that fall risk scales developed for specific clinical settings, such as rehabilitation hospitals, demonstrate stronger construct validity and clinical relevance compared to general instruments. These findings further support the need for oncology‐specific tools that reflect the unique treatment‐related and functional characteristics of cancer patients [18].

In comparison, the reliability study of the Morse Fall Risk Scale reported a Cronbach's alpha value of 0.65 [14], and the Turkish validity and reliability study of the Wilson‐Sims Psychiatric Fall Risk Assessment Scale reported a Cronbach's alpha value of 0.89 [15]. These findings highlight the strong internal consistency of FRAS‐OP; however, higher reliability coefficients alone should not be interpreted as evidence of superior predictive performance.

Based on these results, FRAS‐OP appears to be a promising instrument for identifying oncology‐specific fall risk factors and supporting clinical risk assessment processes. However, since criterion validity was not evaluated using an established fall risk scale and predictive validity based on prospective fall outcomes was not assessed, claims regarding early identification of future falls or fall prevention effectiveness should be interpreted cautiously. Further longitudinal and comparative studies are required to determine the scale's ability to predict actual fall events.

## 5 Conclusion

This study contributes to the literature by developing an original and reliable scale for assessing fall risk in oncology patients. The FRAS‐OP demonstrated high internal consistency and satisfactory construct validity.

Fall Risk Assessment Scale for Oncology Patients provides a structured assessment framework that may support healthcare professionals in identifying potential fall risk factors specific to oncology patients and in planning individualised preventive care strategies. Although the scale shows strong psychometric properties, its effectiveness in predicting fall incidents and reducing fall‐related injuries has not yet been empirically established. Future prospective studies are necessary to evaluate its predictive validity and clinical impact across different settings and patient populations.

## 6 Limitations

This study has some limitations. First, the RMSEA and GFI values were slightly outside the ideal range, indicating the need for further validation studies using different and larger samples. Second, the study was conducted in a single university hospital, which limits the generalizability of the findings due to restricted sample diversity. Additionally, criterion validity could not be assessed because the established fall risk scale was administered concurrently, and the predictive validity analyses based on prospective fall outcomes were not performed. These limitations highlight the need for future multi‐center and longitudinal studies to further evaluate the scale's validity and applicability in diverse oncology populations.

## Author Contributions

Sibel Serçe, Soner Berşe, Özlem Ovayolu, and Nimet Ovayolu made substantial contributions to the conception or design of the work, and to the acquisition, analysis, or interpretation of data. Sibel Serçe, Soner Berşe, Özlem Ovayolu, and Nimet Ovayolu drafted the work or revised it critically for important intellectual content. Özlem Ovayolu provided final approval of the version to be published. Sibel Serçe, Soner Berşe, Özlem Ovayolu, and Nimet Ovayolu agree to be accountable for all aspects of the work in ensuring that questions related to the accuracy or integrity of any part of the work are appropriately investigated and resolved.

## Funding

The authors have nothing to report.

## Disclosure

The authors have nothing to report.

## Ethics Statement

Ethics committee approval (protocol code: 2022/213) and institutional permission were obtained from the Non‐Interventional Research Ethics Committee of the University of Gaziantep University, Turkey.

## Conflicts of Interest

The authors declare no conflicts of interest.

## Data Availability

The data that support the findings of this study are available on request from the corresponding author. The data are not publicly available due to privacy or ethical restrictions.
